# Myogenic IL-6 Mediates Myofiber Type Transition in Porcine Skeletal Muscle Satellite Cells via the STAT3/*myh7* Pathway

**DOI:** 10.3390/vetsci13090927

**Published:** 2026-09-09

**Authors:** Rui Cao, Hui Wei, Chao Li, Qiuming Deng, Chongfan Du, Ruiyi Lin, Tianfang Xiao, Weimin Lin

**Affiliations:** Engineering Research Center for Animal Breeding and Sustainable Production, College of Animal Science, Fujian Agriculture and Forestry University, Fuzhou 350002, China; 12406009004@fafu.edu.cn (R.C.); 12506009013@fafu.edu.cn (H.W.); 52406009020@fafu.edu.cn (C.L.); 52506009023@fafu.edu.cn (Q.D.); 12306009010@fafu.edu.cn (C.D.); linruiyi@fafu.edu.cn (R.L.); tfxiao@163.com (T.X.)

**Keywords:** pig, skeletal muscle, myofiber type, IL-6, STAT3

## Abstract

Myofiber type determines pork quality, with type I fibers conferring superior traits via myosin heavy chain 7 (Myh7). As a crucial myokine, interleukin-6 (IL-6) plays important roles in skeletal muscle development, whether it participates in *myh7* transcription and type I myofiber differentiation remains unclear. To address this, we compared Putian (adipose-type) and DLY (lean-type) pigs through in vivo myofiber typing and in vitro satellite cell (SC) cultures, supplemented by ChIP-PCR and dual-luciferase reporter assays to investigate molecular mechanisms. Our results showed that Putian pigs exhibited significantly higher proportions of type I myofibers, *myh7* mRNA and Myh7 protein expression, and IL-6 levels than DLY pigs. In vitro, IL-6 treatment promoted SC differentiation toward type I myofibers. Mechanistically, STAT3 bound directly to the *myh7* promoter in a phosphorylation-dependent manner to enhance its transcription, as confirmed by ChIP-PCR and dual-luciferase reporter assays. STAT3 was required for IL-6-induced *myh7* upregulation, and STAT3 inhibition abrogated this effect. Collectively, these findings demonstrate that muscle-derived IL-6 promotes type I myofiber differentiation by activating STAT3 phosphorylation and *myh7* transcription, providing a candidate target for improving pork quality through selective breeding or nutritional intervention.

## Simple Summary

The quality of pork depends largely on the composition of myofiber types. Type I myofibers are linked to better meat characteristics, such as improved intramuscular fat deposition and water-holding capacity. In this study, we compared two pig breeds: Putian pigs (a Chinese indigenous breed) and DLY pigs (a conventional lean breed). We found that Putian pigs have more type I myofibers and higher levels of a muscle-derived signal protein called interleukin-6 (IL-6). In laboratory experiments with pig muscle satellite cells, adding IL-6 guided these cells to develop into type I myofibers rather than other myofiber types. Further investigation revealed that IL-6 works by activating a key molecular switch called STAT3, which turns on the *myh7* gene, a master controller of type I myofiber formation. Blocking STAT3 activity prevented IL-6 from promoting type I myofibers. These findings uncover a new mechanism by which muscle satellite cells control their myofiber identity and suggest that targeting the IL-6/STAT3/*myh7* pathway could help breeders or nutritionists improve pork quality through selective breeding or dietary strategies.

## 1. Introduction

Skeletal muscle, the primary component of pork and a major source of animal protein, is composed of myofibers—the largest cells in mammals, accounting for over 75% of skeletal muscle mass [1]. Key myofiber characteristics, including type, number, diameter, and density, are critical determinants of pork quality traits such as meat color, pH, drip loss, water-holding capacity, and intramuscular fat (IMF) content [2,3].

Postmortem meat quality is directly influenced by the proportion of skeletal myofiber types. Based on metabolic and contractile properties, myofibers are classified into type I (oxidative) and type II (glycolytic) fibers [4,5]. During growth and development, myofiber types can undergo transitions, characterized by the replacement of one myosin heavy chain (MyHC) isoform with another [6]. Based on MyHC isoforms, myofibers are categorized into four types: MyHCI, MyHCIIa, MyHCIIx, and MyHCIIb. At birth, porcine muscle consists almost entirely of type I myofibers; subsequently, influenced by various intrinsic and extrinsic factors, a gradual shift occurs from type I toward type II myofibers following the sequential pattern I ↔ IIa ↔ IIx ↔ IIb [7].

The interconversion of myofiber types is regulated by multiple signaling pathways. Among these, the AMPK/PGC-1α pathway plays a key role in muscular energy metabolism and is closely associated with fiber type transformation [8,9]. Although AMPK/PGC-1α is a central hub regulating oxidative fiber specification, its activity is tightly coupled with cellular energy status. Interestingly, Chinese indigenous pig breeds, such as Putian pigs, exhibit a higher proportion of type I fibers along with increased intramuscular fat content, suggesting the existence of AMPK-independent regulatory mechanisms. Recent functional genomic and molecular genetic studies have identified Myh7 as a key mediator regulating the differentiation of type I myofibers in porcine skeletal muscle [10].

Skeletal muscle damage, which can result from various factors including exercise, is primarily repaired by skeletal muscle satellite cells (SCs). Recent studies indicate that exercise induces local pH shifts in muscle tissue, leading to succinate release, which promotes the differentiation of non-muscle-derived fibroblasts and contributes to the regulation of myofiber type composition [11,12].

Muscle contraction during exercise also stimulates the secretion of myokines. Among these, interleukin-6 (IL-6) is notably the most responsive, with serum levels increasing nearly 100-fold after intense exercise [13]. While other myokines such as IL-4 and IL-15 are also involved in muscle regeneration [14,15], IL-6 is unique in that it is directly induced by muscle contraction and acts in a hormone-like manner to regulate systemic metabolism, making it a prime candidate for mediating exercise-induced myofiber remodeling [16,17]. Aerobic exercise has been shown to reduce type II myofiber proportion while increasing type I myofibers in human skeletal muscle, thereby improving muscular endurance [18]. Consistent with this, IL-6 knockout mice fed a high-fat diet exhibit an elevated proportion of type II myofibers [19]. However, the mechanism through which IL-6 influences myofiber type transformation in porcine skeletal muscle remains unclear.

Therefore, this study aimed to elucidate the molecular mechanism by which muscle-derived IL-6 regulates skeletal myofiber type conversion in pigs, offering a clearer understanding of myokine-mediated control over myofiber phenotype.

## 2. Materials and Methods

### 2.1. Animals

Crossbred (Duroc × Landrace × Yorkshire, DLY) and Putian pigs at two developmental stages, 7-day-old neonates and 180-day-old market-age individuals, were sourced from Fujian Yongcheng Farming and Animal Husbandry Co., Ltd. (Fuzhou, China) and Putian Xianglixiang Black Pig Development Co., Ltd. (Putian, China), respectively. Neonatal piglets were used for primary cell isolation, whereas postpubertal pigs were selected for myofiber type quantification.

### 2.2. Cell Isolation, Culture and Differentiation

According to the regulations of the Experimental Animal Welfare Professional Committee of the Chinese Association for Laboratory Animal Sciences, 7-day-old piglets were subcutaneously injected with Zoletil-50 (10 mg/kg). After deep anesthesia was achieved, the animals were rapidly exsanguinated and sacrificed. Satellite cells (SCs) were isolated from the longissimus dorsi muscle of piglets. Muscle tissue was minced into 1–2 mm^3^ pieces and digested with 0.1% collagenase type I (Gibco, Shanghai, China) at 37 °C for 90 min with 50 rpm shaking. The digest was sequentially filtered through 200-μm and 40-μm nylon meshes, and the filtrate was centrifuged at 1000 rpm for 5 min. The pellet was resuspended in proliferation medium consisting of RPMI 1640 medium (Gibco, Shanghai, China) supplemented with 10% fetal bovine serum (Umedium, Hefei, China), 0.5% chicken embryo extract (Gemini, Wuhan, China), 1% GlutaMax (Gibco, Shanghai, China), 1% non-essential amino acids (Gemini, Wuhan, China), 2% penicillin–streptomycin (Gemini, Wuhan, China), and 5 ng/mL basic fibroblast growth factor (CHAMOT, Shanghai, China). To remove fibroblasts, cells were pre-plated on uncoated dishes for 1 h, and the non-adherent enriched SCs were then seeded onto collagen-coated plates. Cells were cultured at 37 °C in a humidified 5% CO_2_ atmosphere, with medium replacement every 48 h.

Myogenic differentiation was induced for 2 days using differentiation medium (Dulbecco’s Modified Eagle’s Medium/Ham’s F-12 High Glucose supplemented with 10% fetal bovine serum, 5% horse serum, and 2% penicillin–streptomycin), with medium changes every 48 h. Differentiation efficiency was confirmed by the formation of myosin heavy chain-positive myotubes.

### 2.3. Plasmid and Small Interfering RNA Transfection

SCs were seeded in six-well plates at a density of 2 × 10^5^ cells/well and grown to approximately 80% confluence before transfection. Transfections were performed using Lipofectamine^TM^ 3000 (Invitrogen, Shanghai, China) according to the manufacturer’s instructions. For plasmid transfection, each well received 2000 ng of recombinant plasmid mixed with 5 μL Lipofectamine^TM^ 3000 in 250 μL Opti-MEM. For siRNA transfection, 6 μL of siRNA (final concentration 60 nM) was complexed with 5 μL Lipofectamine^TM^ 3000 in the same volume of Opti-MEM. After 8 h, the transfection mixture was replaced with complete proliferation medium. Cells were harvested for protein or RNA analysis within 48 h post-transfection. Negative controls included empty pcDNA3.1 plasmid or non-targeting siRNA (si-NC), transfected under identical conditions. siRNA sequences and gene-specific primers are provided in Supplementary Table S1.

### 2.4. IL-6 Recombinant Protein and STAT3 Inhibitor Treatment

SCs were seeded in six-well plates at 2 × 10^5^ cells/well and grown to approximately 80% confluence. For IL-6 treatment, recombinant IL-6 protein (HY-P73220, MCE, Shanghai, China, >95% purity) was dissolved in PBS containing 0.1% BSA and added to the culture medium at final concentrations of 0, 50, and 100 ng/mL for 24 h. For STAT3 inhibition, cells were pretreated with the STAT3-specific inhibitor Angoline (HY-N7674, MCE, Shanghai, China) at 50 ng/mL for 2 h, followed by co-treatment with or without 100 ng/mL IL-6 for an additional 24 h. Cells were then harvested for RNA and protein extraction.

### 2.5. Histological Assay

Histochemical identification of myofiber types in the longissimus dorsi (collected from 180-day-old castrated males, *n* = 6 per breed) was performed using an ATPase staining kit (G2380, Solarbio, Beijing, China). Briefly, muscle samples were frozen in isopentane near its freezing point and sectioned at 20 µm. Sections were sequentially incubated in acid pre-incubation solution (pH 4.2, 5 min), ATPase incubation solution (30 min), 1% CaCl_2_ (3 min), cobalt solution (3 min), and sulfide working solution (1 min). After rinsing and dehydration, slow myofibers were identified by the presence of ATPase-positive black precipitate. For each animal, five randomly selected fields per section (three sections per muscle) were analyzed, and at least 200 fibers were counted per field. The proportion of type I fibers was calculated as (number of type I fibers/total fibers) × 100%.

### 2.6. RNA Extraction and qPCR

Total RNA was extracted from cells and muscle tissue using a Cell/Tissue Total RNA Kit (YEASEN, Shanghai, China). cDNA was synthesized following the manufacturer’s instructions. Quantitative real-time PCR (qPCR) was performed using Hieff UNICON^®^ Universal Blue qPCR SYBR Green Master Mix (YEASEN, Shanghai, China) on a QuantStudio 5 Real-Time PCR System (Applied Biosystems, Shanghai, China). All reactions were run in triplicate with no-template controls. Relative mRNA expression was calculated via the 2^−^^ΔΔCt^ method, using *GAPDH* as the endogenous control, its expression stability under our culture conditions was confirmed (Ct variation < 0.5). Amplification efficiency for each primer pair was determined by a standard curve generated from five serial 10-fold dilutions of pooled cDNA (ranging from 1:1 to 1:10^4^). The efficiency (E) was calculated as E = 10^−1/slope^ − 1, and all primer pairs exhibited efficiencies between 90% and 110% (Supplementary Table S1), fulfilling the prerequisite for the ΔΔCt quantification method.

### 2.7. Protein Extraction and Western Blot

Total protein was extracted from cells and muscle tissue using RIPA buffer (Beyotime, Shanghai, China) containing protease and phosphatase inhibitors (1 mM PMSF). Protein concentration was determined using a BCA kit (Beyotime, Shanghai, China). Equal amounts of protein (15 µg per sample) were separated by 12% SDS-PAGE and transferred to 0.45 μm PVDF membranes (Millipore, Billerica, MA, USA). Membranes were blocked with 5% BSA for 2 h at room temperature and incubated overnight at 4 °C with primary antibodies: anti-Myh4 (BF-F3, DSHB, lowa City, IA, USA, 1:1000), anti-Myh7 (BA-D5, DSHB, lowa City, IA, USA, 1:1000), anti-STAT3 (AF1492, Beyotime, Shanghai, China, 1:1000), anti-phospho-STAT3 (Tyr705, AF5941, Beyotime, Shanghai, China, 1:1000), and anti-GAPDH (ABclonal, Wuhan, China, 1:10,000). After washing, membranes were incubated with HRP-conjugated goat anti-rabbit IgG (11203, ZEB-BIO, Chengdu, China, 1:1000) for 2 h at room temperature. Protein bands were visualized and quantified using an Odyssey^®^ DLx imaging system (LI-COR Biosciences, Lincoln, NE, USA). Additionally, for immunoblotting, we verified key results by total protein normalization, which yielded consistent trends.

### 2.8. Immunofluorescence

Cells cultured on coverslips were fixed with 4% paraformaldehyde for 15 min at room temperature, permeabilized with 0.1% Triton X-100 on ice, and blocked with 5% BSA at 4 °C. Cells were incubated overnight with primary antibodies: anti-Myh7 (A7564, ABclonal, Wuhan, China, 1:50) and anti-Myh4 (BF-F3, DSHB, lowa City, IA, USA, 1:50). After washing, cells were incubated with ABflo 488-conjugated goat anti-rabbit IgG (AS053, ABclonal, Wuhan, China, 1:1000) and ABflo 594-conjugated goat anti-mouse IgG (AS053, ABclonal, Wuhan, China, 1:1000) for 1 h at room temperature. Nuclei were counterstained with DAPI (P0131, Beyotime, Shanghai, China). Images were acquired using a fluorescence microscope.

### 2.9. Dual-Luciferase Reporter Assay

The porcine *myh7* promoter region (−1500 to +100 bp relative to the transcription start site) was amplified from DLY pig genomic DNA and cloned into the pGL3-Basic vector (Promega, Madison, WI, USA). Three putative STAT3 binding sites within this region were mutated individually or in combination using a site-directed mutagenesis kit (YEASEN, Shanghai, China). SCs were seeded in 24-well plates at 5 × 10^4^ cells/well and co-transfected with 500 ng of pGL3-Myh7 (wild-type or mutant), 50 ng of pRL-TK Renilla luciferase control vector, and 500 ng of pcDNA3.1-STAT3 or empty vector using Lipofectamine™ 3000. After 48 h, firefly and Renilla luciferase activities were measured using the Dual-Luciferase Reporter Gene Assay Kit (RG027, Beyotime, Shanghai, China). Firefly signals were normalized to Renilla activity. Each experiment included six independent biological replicates (each with three technical replicates).

### 2.10. Chromatin Immunoprecipitation Assay PCR (ChIP-PCR)

ChIP assays were performed using a commercial kit (P2078, Beyotime, Shanghai, China). Differentiated SCs (day 2 of differentiation) were crosslinked with 1% formaldehyde for 10 min at room temperature. Glycine was added to stop crosslinking. Cells were lysed and sonicated using a VCX750 sonicator (Sonics, Newtown, CT, USA) at 30% amplitude for 15 cycles (30 s on, 30 s off) to fragment genomic DNA to 200–1000 bp. For immunoprecipitation, 100 µL of cell lysate was incubated overnight at 4 °C with 2 µg of anti-STAT3 antibody (12382-1-AP, Proteintech, Rosemont, IL, USA) or normal rabbit IgG (as negative control) (AS053, ABclonal, Wuhan, China). Antibody–protein complexes were precipitated with 60 µL of Protein A Agarose/Salmon Sperm DNA slurry for 2 h at 4 °C, followed by sequential washes. Enriched DNA was purified and analyzed by PCR using primers specific to the *myh7* promoter region (sequences in Supplementary Table S1). PCR products were separated by agarose gel electrophoresis and visualized using a Tanon 1600 gel documentation system (Tanon, Shanghai, China).

### 2.11. Bioinformatic Analysis

The transcriptional regulatory elements of the porcine *myh7* promoter (upstream 2000 bp from the transcription start site) were predicted using the Promoter 2.0 online tool (http://www.cbs.dtu.dk/services/Promoter/, URL (accessed on 1 April 2026)). Putative transcription factor binding sites within the promoter region were subsequently identified via the JASPAR 2024 database (http://jaspar.genereg.net/, URL (accessed on 15 April 2026)) with a relative profile score threshold of >85%.

### 2.12. Statistical Analysis

All statistical analyses were performed using SPSS Statistics 25.0 (IBM Corp., Armonk, NY, USA). Data are presented as mean ± standard error of the mean (SEM). A minimum of three independent biological replicates (n = 3, unless otherwise stated) was used for each experiment. For comparisons between two groups, two-tailed Student’s *t*-test were used. For comparisons involving more than two groups, the one-way ANOVA was used. *p* < 0.05 was considered statistically significant. The sample size for each experiment is indicated in the corresponding figure legends.

## 3. Results

### 3.1. Myofiber Type Composition Differs Between Putian and DLY Pigs

**Figure 1:**
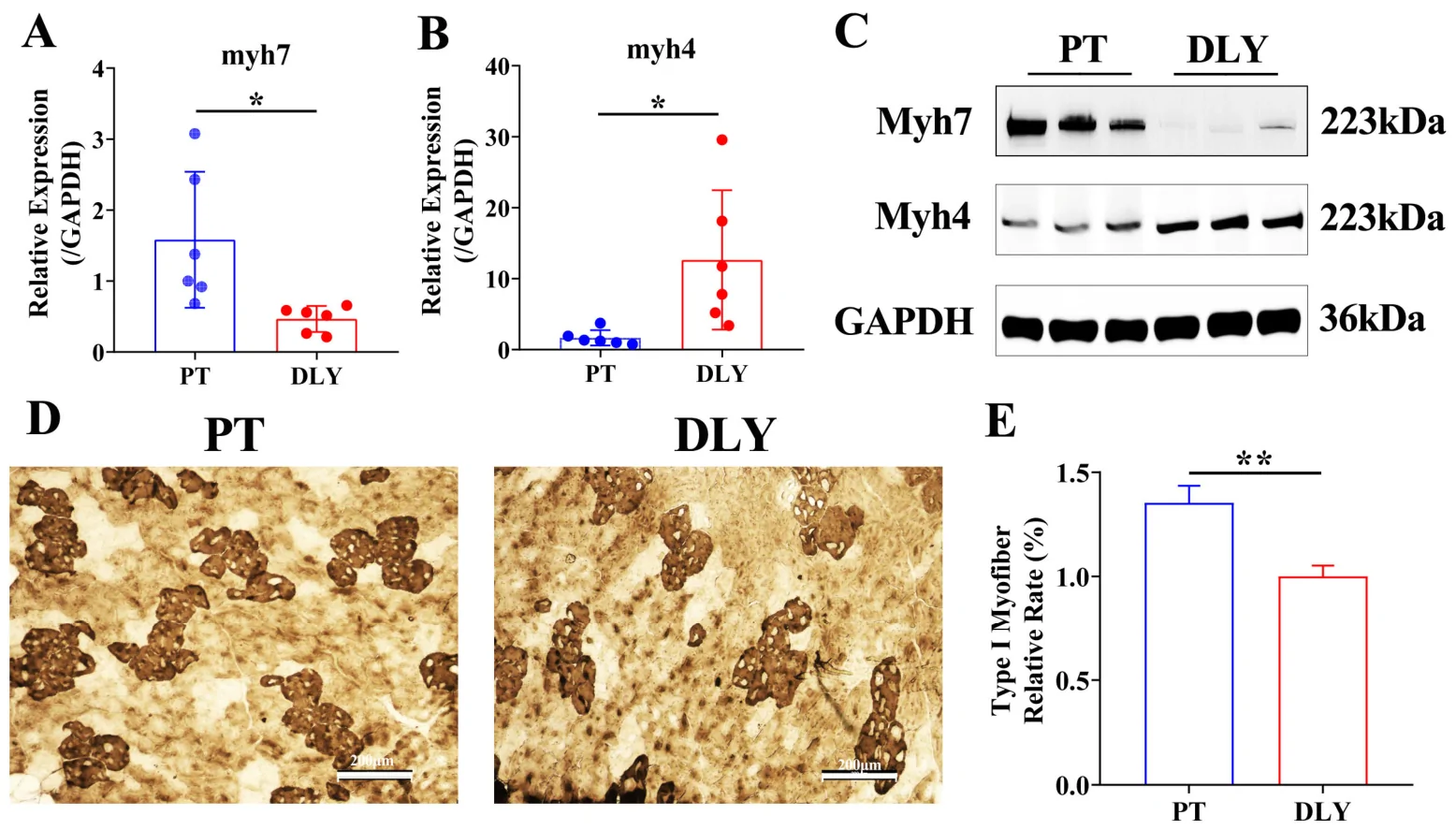
Comparison of the longissimus dorsi muscle between Putian pigs and DLY pigs. (**A**,**B**) mRNA expression levels of *myh4* (**A**) and *myh7* (**B**) in the longissimus dorsi muscle of Putian pigs and DLY pigs, determined by qPCR. Data are normalized to *GAPDH*. (**C**) Representative Western blot images (upper panel) and quantitative analysis (lower panel) of Myh4 and Myh7 protein levels. GAPDH was used as loading control. (**D**,**E**) Representative images of ATPase staining of the longissimus dorsi muscle (**D**) and the corresponding quantitative analysis of type I myofiber proportion (**E**). Black precipitate indicates type I fibers. Scale bar: 200 μm. n = 6 per breed. Data are presented as mean ± standard error of the mean (SEM). PT, Putian pig; DLY, Duroc × Landrace × Yorkshire pig. * *p* < 0.05, ** *p* < 0.01.

To investigate differences in myofiber type composition between Putian and DLY pigs, longissimus dorsi muscle samples from 180-day-old castrated males were collected. ATPase histochemical staining, qPCR, and Western blot were performed to assess myofiber type-specific markers. The mRNA levels of *myh7*, a marker for type I fibers, were significantly higher (approximately 3.3-fold, *p* < 0.05) in Putian pigs than in DLY pigs (Figure 1A). Conversely, *myh4* (type IIb myofiber marker) expression was significantly lower (by ~86.8%, *p* < 0.05) in Putian pigs (Figure 1B). Western blot analysis showed that Myh7 protein was readily detectable in Putian pigs but was extremely low or undetectable in DLY pigs, whereas Myh4 protein showed the opposite pattern (Figure 1C). ATPase staining confirmed a significantly greater proportion of type I myofibers in the longissimus dorsi muscle of Putian pigs compared to DLY pigs (*p* < 0.01, Figure 1D,E).

### 3.2. IL-6 Promotes Myogenic Differentiation Toward Type I Myofibers in Porcine SCs

**Figure 2:**
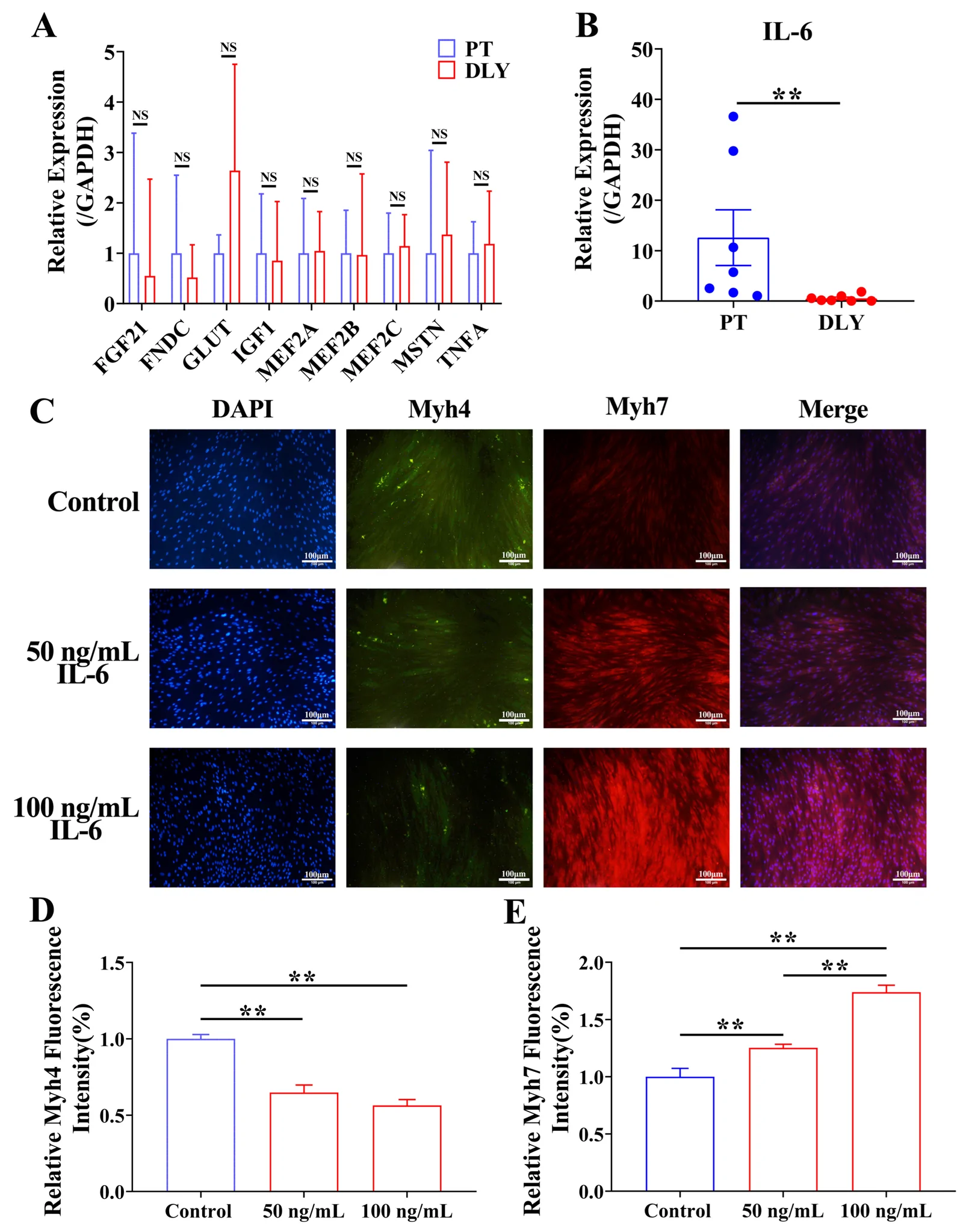
IL-6 promotes myogenic differentiation toward type I myofibers in porcine SCs. (**A**) mRNA expression levels of indicated myokines in the longissimus dorsi muscle of Putian pigs and DLY pigs (n = 7 per breed). (**B**) IL-6 mRNA levels from panel A shown separately. (**C**) Representative immunofluorescence images of differentiated SCs treated with 0, 50, or 100 ng/mL recombinant IL-6 for 48 h. Myh7 (type I, red), Myh4 (type IIb, green), DAPI (blue) (n = 3 per group). Scale bar: 100 μm. (**D**,**E**) Quantitative analysis of the proportion of type IIb (Myh4-positive) myotubes (**D**) and type I (Myh7-positive) myotubes (**E**). Data are presented as mean ± standard error of the mean (SEM). PT, Putian pig; DLY, Duroc × Landrace × Yorkshire pig. Scale bar: 100 μm. NS, *p* > 0.05, ** *p* < 0.01.

To identify the key regulatory factors involved in muscle fiber type differentiation, the mRNA expression levels of various muscle-specific factors—FGF21, FNDC5, GLUT4, IGF1, MEF2A, MEF2B, MEF2C, MSTN, TNF-α, and IL-6—that were detectable at appreciable levels were compared in the longissimus dorsi muscle tissues of Putian pigs and DLY (Duroc × Landrace × Yorkshire) pigs. Among these, only *IL-6* mRNA levels were significantly higher (approximately 23.7-fold, *p* < 0.05,) in the longissimus dorsi of Putian pigs compared to DLY pigs (Figure 2A,B), suggesting a specific association with type I fiber enrichment.

To determine whether muscle-derived IL-6 influences the differentiation of porcine SCs, DLY SCs were treated with recombinant IL-6 protein (50 or 100 ng/mL) for 24 h, followed by myogenic differentiation induction for 48 h. Immunofluorescence staining for Myh7 (type I) and Myh4 (type IIb) revealed that IL-6 treatment significantly increased the proportion of type I myofibers (from 100.0% in control to 125.3% at 50 ng/mL and 173.9% at 100 ng/mL, *p* < 0.01) and decreased the proportion of type IIb myofibers (from 100% in control to 64.8% at 50 ng/mL and 56.4% at 100 ng/mL, *p* < 0.01) in a dose-dependent manner (Figure 2C–E). These results indicate that IL-6 promotes SC differentiation toward a type I myofiber phenotype.

### 3.3. STAT3 Transcriptionally Upregulates myh7 by Binding to Its Promoter

**Figure 3:**
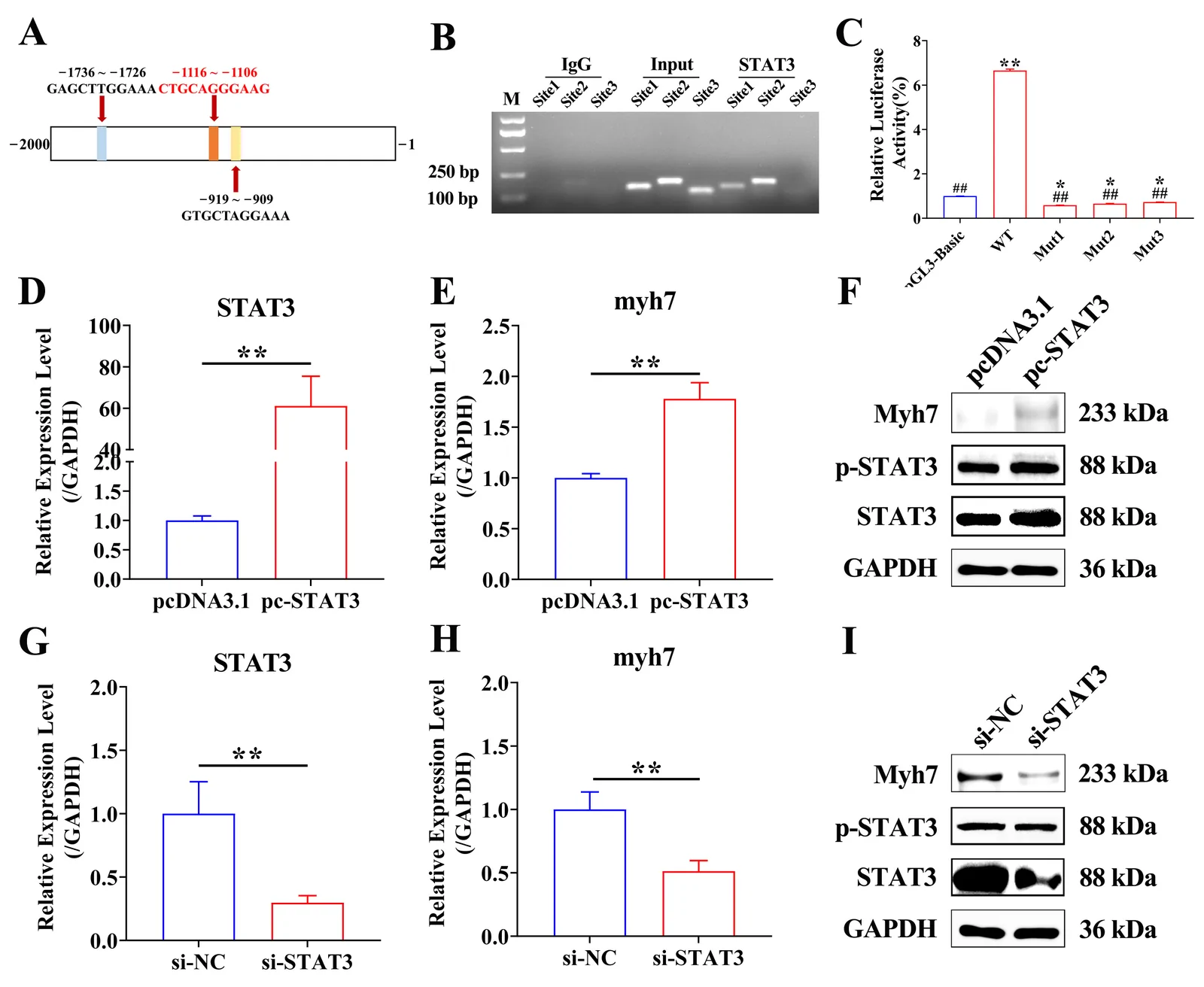
STAT3 transcriptionally upregulates *myh7* by binding to its promoter. (**A**) Schematic representation of STAT3 binding sites (S1–S3) in the porcine *myh7* promoter region. (**B**) ChIP-PCR analysis confirming STAT3 binding to the *myh7* promoter. (**C**) Dual-luciferase reporter assay showing STAT3-mediated transcriptional activation of the porcine *myh7* promoter. (**D**) *STAT3* mRNA levels after STAT3 overexpression in SCs. (**E**,**F**) *myh7* mRNA (**E**) and protein (**F**) levels after STAT3 overexpression in SCs. (**G**) *STAT3* mRNA levels after STAT3 inhibition in SCs. (**H**,**I**) *myh7* mRNA (**H**) and protein (**I**) levels after STAT3 inhibition in SCs. Data are presented as mean ± standard error of the mean (SEM). n = 3 per group. * *p* < 0.05, ** *p* < 0.01 (compared with respective controls). ## *p* < 0.01 indicates significant difference between the wild-type (WT) group and other experimental groups. WT, wild-type sequence of the STAT3 binding motif.

As a key downstream effector of IL-6 signaling, STAT3 functions as a critical transcription factor. Bioinformatics analysis identified three potential STAT3 binding sites (S1: −1736 to −1726, S2: −1116 to −1106, S3: −919 to −909 relative to TSS) within the *myh7* promoter region (Figure 3A). ChIP-PCR assays using differentiated SCs confirmed that STAT3 binds to S1 and S2 but not to S3 under basal conditions (Figure 3B). Dual-luciferase reporter assays in SCs co-transfected with STAT3 expression plasmid showed that mutation of S1–S3 individually significantly reduced STAT3-induced promoter activity (by 41.1%, 34.3% and 26.8%, respectively, *p* < 0.05) (Figure 3C). Overexpression of STAT3 using pcDNA3.1-STAT3 led to a ~61.0-fold increase in *STAT3* mRNA (Figure 3D), an increase in total STAT3 protein and p-STAT3 (Tyr705) levels (Figure 3F). Concomitantly, *myh7* mRNA and Myh7 protein levels were significantly elevated (by 1.8-fold, *p* < 0.01, Figure 3E,F). Conversely, siRNA-mediated knockdown of STAT3 reduced *STAT3* mRNA by 70.2% (Figure 3G), decreased total STAT3 and p-STAT3 protein levels, and significantly downregulated Myh7 expression at both mRNA and protein levels (by 51.3%, *p* < 0.01, Figure 3H,I). Collectively, these results indicate that STAT3, upon phosphorylation-mediated activation, directly binds to the *myh7* promoter to enhance its transcription.

### 3.4. STAT3 Increases Myogenic Differentiation Toward Type I Myofibers in Porcine SCs

**Figure 4:**
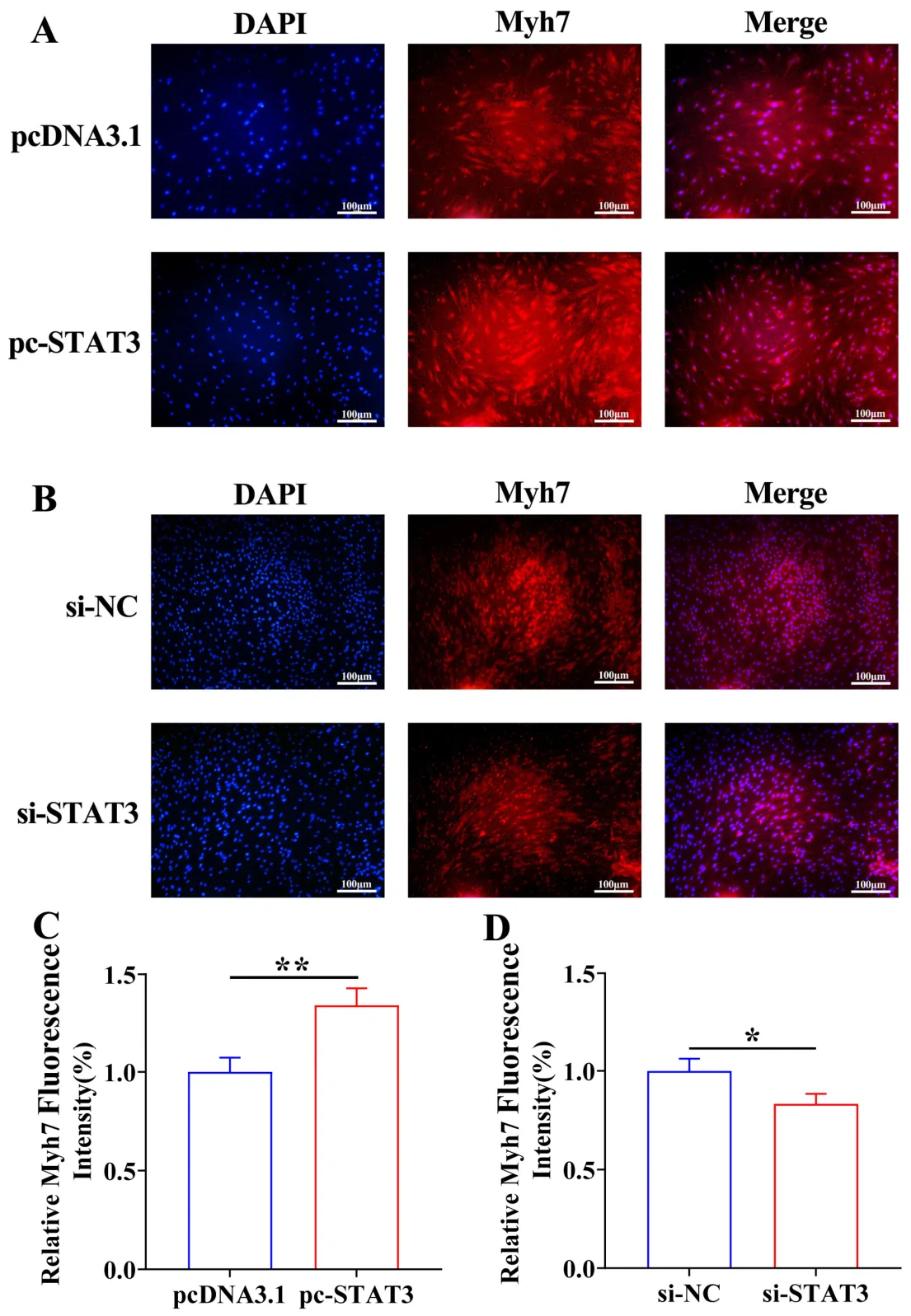
STAT3 increases myogenic differentiation toward type I myofibers in porcine SCs. (**A**,**B**) Immunofluorescence staining of type I and type IIb myofibers after STAT3 overexpression (**A**) and STAT3 inhibition (**B**). (**C**,**D**) Corresponding quantitative analysis of type I myofibers after STAT3 overexpression (**C**) and STAT3 inhibition (**D**). Data are presented as mean ± standard error of the mean (SEM), n = 3 per group. * *p* < 0.05, ** *p* < 0.01.

To investigate the role of STAT3 in myofiber type specification, SCs were transfected with STAT3 overexpression plasmid or STAT3-targeting siRNAs, followed by differentiation induction for 48 h. Myofiber types were assessed by immunofluorescence. Overexpression of STAT3 significantly increased the proportion of Myh7-positive (type I) myotubes from 100.0% in control to 134.2% (*p* < 0.01, Figure 4A,C). In contrast, STAT3 knockdown markedly reduced the type I myofiber population from 100.0% to 83.3% (*p* < 0.05, Figure 4B,D). These findings indicate that STAT3 promotes SC differentiation toward the type I myofiber lineage.

### 3.5. IL-6 Mediates STAT3 Phosphorylation to Promote myh7 Transcription

**Figure 5:**
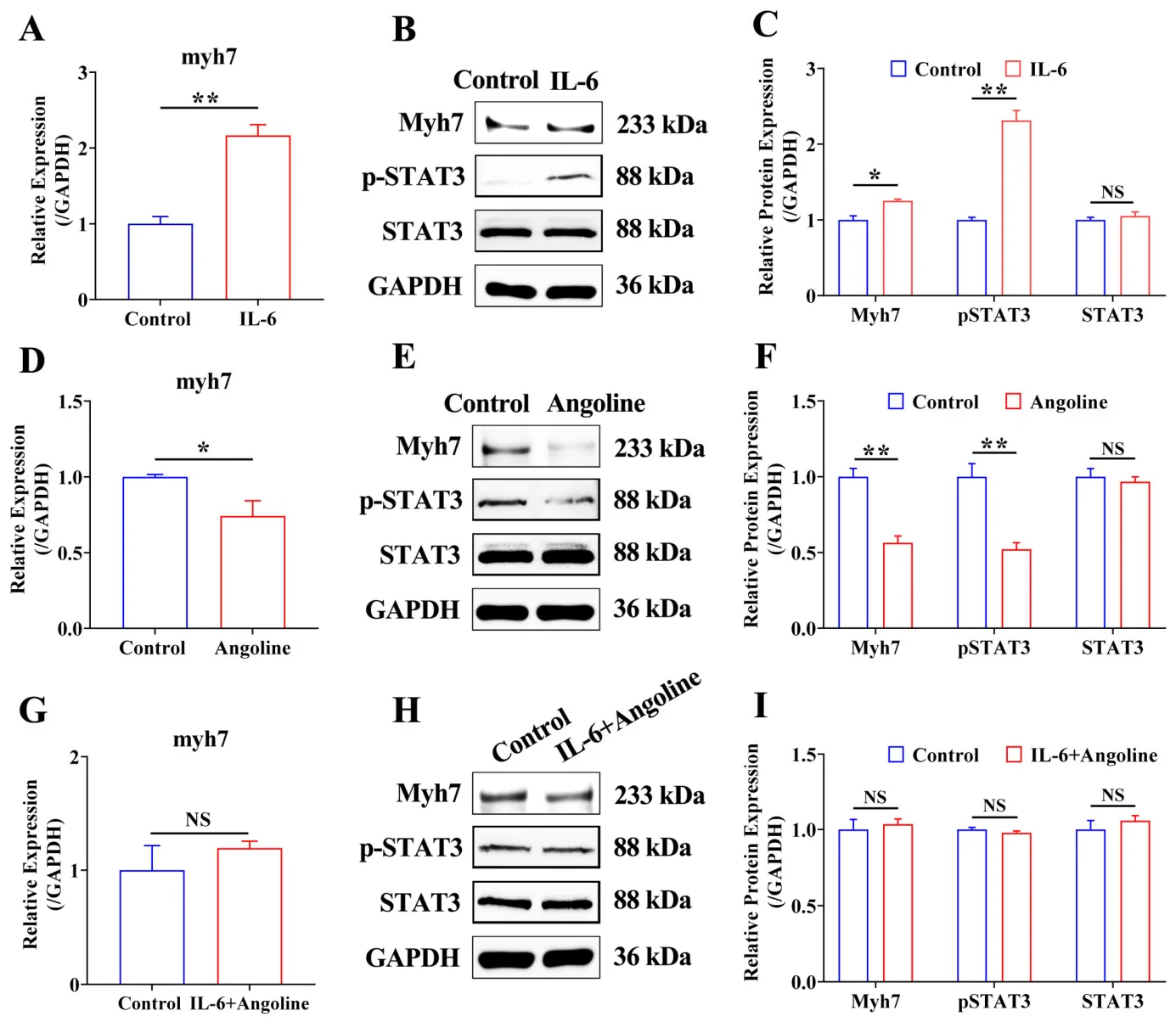
IL-6 mediates STAT3 phosphorylation to promote *myh7* transcription. (**A**) *myh7* mRNA levels after recombinant IL-6 treatment in SCs. (**B**,**C**) Protein levels of Myh7, STAT3, and phosphorylated STAT3 after recombinant IL-6 treatment (**B**), with corresponding quantitative analysis (**C**). (**D**) *myh7* mRNA levels after Angoline treatment in SCs. (**E**,**F**) Protein levels of Myh7, STAT3, and phosphorylated STAT3 after Angoline treatment (**E**), with corresponding quantitative analysis (**F**). (**G**) *myh7* mRNA levels after co-treatment with recombinant IL-6 and Angoline in SCs. (**H**,**I**) Protein levels of Myh7, STAT3, and phosphorylated STAT3 after co-treatment (**H**), with corresponding quantitative analysis (**I**). Data are presented as mean ± standard error of the mean (SEM), n = 3 per group. NS, *p* > 0.05, * *p* < 0.05, ** *p* < 0.01.

To verify whether IL-6 activates *myh7* expression through the STAT3 signaling pathway, SCs were treated with 100 ng/mL IL-6 for 48 h. Both Myh7 mRNA and protein levels were significantly elevated (mRNA: 2.2-fold, *p* < 0.01; protein: 1.3-fold, *p* < 0.05; Figure 5A–C). Meanwhile, IL-6 treatment increased p-STAT3 (Tyr705) protein levels by 2.3-fold (*p* > 0.01) without affecting total STAT3 (*p* > 0.05) (Figure 5B,C). To determine whether STAT3 phosphorylation is required for *myh7* transcriptional activation, SCs were treated with the STAT3 phosphorylation inhibitor Angoline (50 ng/mL) for 48 h. Angoline treatment markedly reduced p-STAT3 levels (by 47.9%, *p* < 0.01) without affecting total STAT3 (*p* > 0.05) and concomitantly suppressed both Myh7 mRNA and protein expression (mRNA: by 74.2%; protein: by 43.3%; *p* < 0.01, Figure 5D–F). To examine whether Angoline blocks IL-6-mediated *Myh7* transcription, SCs were co-treated with IL-6 and Angoline. Myh7 mRNA and protein levels in the co-treatment group were not significantly different from those in the control group (*p* > 0.05, Figure 5G–I), demonstrating that Angoline abrogates IL-6-induced upregulation of *myh7 gene*. Thus, phosphorylated STAT3 is a key mediator of IL-6-driven Myh7 expression. Collectively, these findings indicate that IL-6 promotes *myh7* transcription through the STAT3 signaling pathway.

## 4. Discussion

Myofiber differentiation is a complex physiological process characterized primarily by the sequential transition of MyHC isoforms (I ↔ IIa ↔ IIx ↔ IIb) [7]. In this study, we assessed myofiber composition in the longissimus dorsi, a representative type II myofiber-rich muscle, of Putian and DLY pigs. ATPase staining and qPCR analysis revealed that type I myofiber content was significantly higher in Putian pigs than in DLY pigs (Figure 1), consistent with previous reports that Chinese indigenous pigs exhibit greater abundance of type I myofibers [20]. Moreover, IL-6 expression was also elevated in Putian pigs (Figure 2B), suggesting a positive association between muscle IL-6 levels and type I fiber enrichment. It is worth noting that Myh7 protein was barely detectable in DLY pigs despite detectable mRNA, suggesting possible post-transcriptional regulation or rapid protein turnover in this breed.

As a classic myokine, IL-6 plays a critical role in regulating satellite cell hypertrophy and exerts pleiotropic effects in skeletal muscle, including promoting lipolysis and enhancing tissue energy uptake [21,22,23]. IL-6 induces STAT3 phosphorylation, which in turn promotes MyoD expression and facilitates myotube differentiation [24,25]. Studies have shown that IL-6 levels and STAT3 phosphorylation are significantly elevated after exercise, accompanied by increased expression of downstream targets such as c-MYC, c-FOS, and SOCS3, highlighting the critical role of the JAK/STAT signaling pathway in muscle regeneration [26]. Our study further demonstrates that IL-6 not only regulates porcine SC differentiation but also specifically mediates their differentiation toward type I myofibers (Figure 2). This finding aligns with observations in humans that exercise-induced elevation of IL-6 promotes a shift toward type I myofibers [18]. Notably, the primary SCs used in this study were isolated from 7-day-old piglets, whose muscles consist almost entirely of type I myofibers at birth [7]. To exclude the possibility that these SCs inherently favor type I differentiation, we characterized their intrinsic differentiation potential and confirmed that they possess the capacity to differentiate into both type I and type IIb fibers, with no inherent bias. IL-6 treatment significantly altered the differentiated myofiber composition (Figure 2C–E), further supporting its critical role in regulating myofiber type specification.

IL-6 exerts its regulatory effects primarily through two pathways: one involves binding to the membrane-bound IL-6 receptor (IL-6R), activating JAK and inducing STAT3 phosphorylation at Y705 [27]; the other involves binding to the gp130 glycoprotein, leading to downstream JAK/STAT3 activation [28,29]. It should be noted that, in addition to this classical pathway, IL-6 can also signal through a trans-signaling pathway, which requires initial binding to the soluble interleukin-6 receptor (sIL-6R). The resulting complex subsequently associates with the gp130, activating the downstream JAK/STAT3 pathway [30,31]. The key distinction between these two signaling modes lies in the differential distribution of IL-6 receptors across cell types. While the membrane-bound IL-6 receptor is expressed on a few cell types, for example, the immune system, hepatocytes and some epithelial cells [32,33], the trans-signaling pathway is active in many different cell types [34]. Importantly, both pathways converge on activation of the JAK/STAT3 axis, with the critical outcome being the induction of STAT3 phosphorylation, which in turn drives transcriptional activation of target genes. Hence, STAT3 serves as a key transcription factor. In our experiments, p-STAT3 levels showed consistent changes with *myh7* gene expression; STAT3 knockdown reduced both p-STAT3 and Myh7, while STAT3 overexpression increased both, confirming that the transcriptional activation function of STAT3 depends on its phosphorylation status (Figure 3 and Figure 4). Moreover, bioinformatic predictions revealed no STAT3 binding sites in the *Myh4* promoter region, suggesting that STAT3 specifically regulates *myh7* transcription rather than broadly activating markers of various muscle fiber types, thereby precisely modulating myofiber differentiation in SCs.

To further elucidate the transcriptional regulation of *myh7* by IL-6 via STAT3 phosphorylation, this study employed Angoline, a STAT3 phosphorylation inhibitor. The results confirmed that IL-6 enhances STAT3 phosphorylation in SCs, thereby activating its targeted binding to the *myh7* gene promoter (Figure 5). One point of caution is that basal p-STAT3 levels varied moderately between experiments, likely due to differences in cell confluence, passage number, or subtle culture stress; however, the consistent direction of change upon IL-6 or Angoline treatment supports our conclusions.

We acknowledge several limitations. First, the comparison between Putian and DLY pigs only indicates an association, not causality; additionally, since we did not detect breed-specific mutations in the *myh7* gene regulatory regions, the observed differences in IL-6 and *myh7* expression may also involve epigenetic modifications (e.g., DNA methylation, histone acetylation) or environmental factors (e.g., diet, rearing conditions), which warrant further investigation. Second, the use of neonatal SCs, while offering high experimental tractability, may not fully recapitulate the behavior of adult SCs. Third, the mechanistic experiments rely heavily on in vitro systems; in vivo validation (e.g., via intramuscular IL-6 injection or muscle-specific STAT3 knockout in pigs) would be needed to establish physiological relevance. Fourth, we used GAPDH as a single reference gene for qPCR and as a loading control for Western blot. Although GAPDH is widely used in porcine muscle fiber typing studies [35,36,37], its expression can vary with muscle fiber type and metabolic state. To mitigate this concern, we pre-verified that *GAPDH* Ct values showed low variability (CV < 0.5) across our experimental conditions and that its amplification efficiency was within the acceptable range. Nevertheless, we recognize that the use of a single housekeeping gene may not fully account for all biological variations; future studies should incorporate additional reference genes (e.g., ACTB, TUBB) and/or total protein normalization for Western blot to further consolidate the findings. Nevertheless, our findings provide a clear molecular framework for future studies.

**Figure 6:**
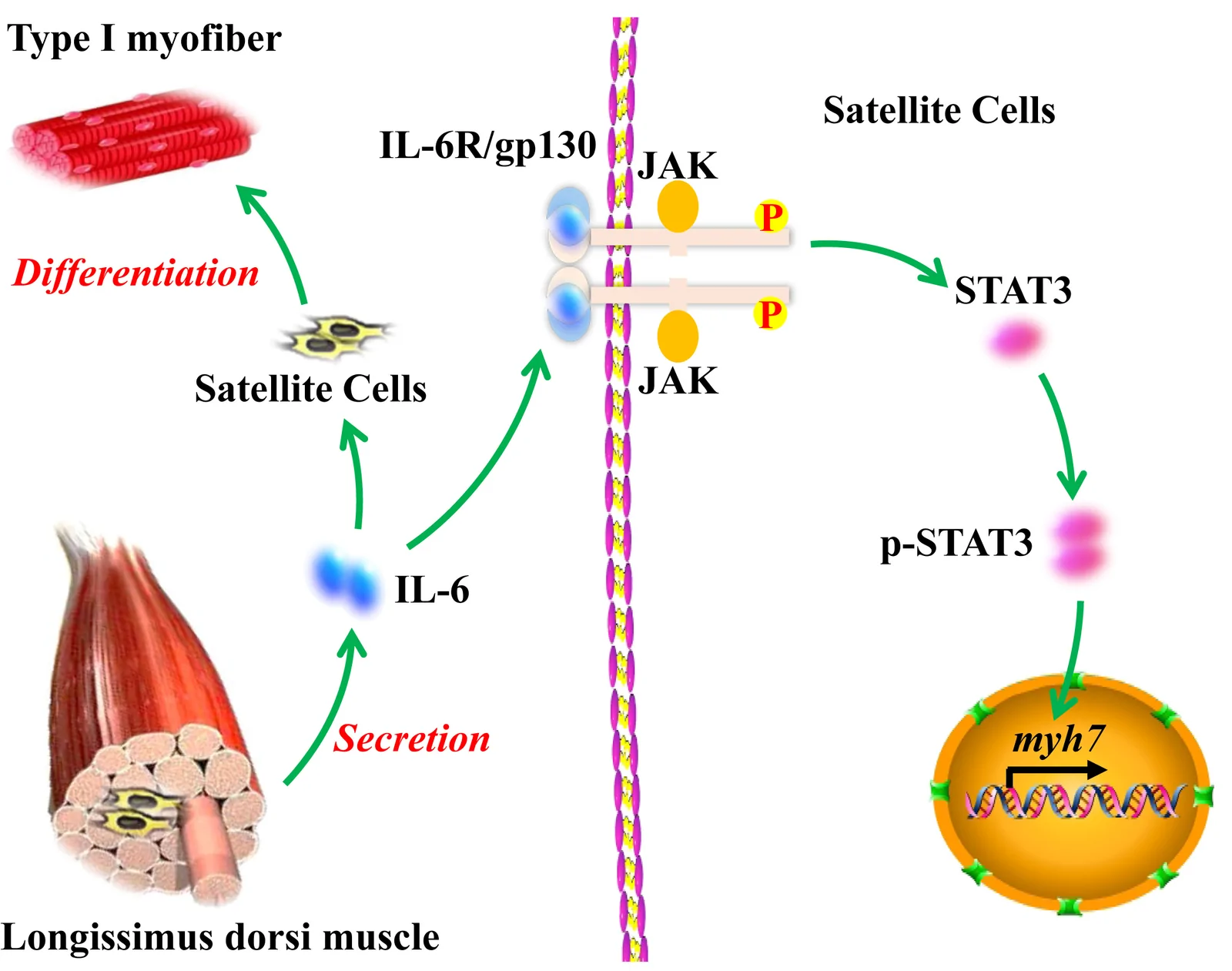
Schematic diagram of the regulatory mechanism by which muscle-derived IL-6 promotes type I myofiber differentiation in satellite cells (SCs) via the STAT3/*myh7* axis. Muscle-derived IL-6 activates the JAK/STAT3 signaling pathway, leading to increased STAT3 phosphorylation. Phosphorylated STAT3 binds to the *myh7* promoter to induce its transcription, thereby enhancing the differentiation of porcine SCs toward type I myofibers. This regulatory axis ultimately governs myofiber type differentiation in porcine skeletal muscle.

In summary, this study demonstrates that muscle-derived IL-6 plays a critical regulatory role in determining the myofiber type differentiation of SCs by activating STAT3 phosphorylation and facilitating its transcriptional function, which mediates *myh7* expression and promotes differentiation toward type I myofibers (Figure 6). These findings provide a mechanistic basis for improving pork quality through targeted manipulation of the IL-6/STAT3/*myh7* axis.

## Data Availability

The original contributions presented in this study are included in the article/Supplementary Material. Further inquiries can be directed to the corresponding author.

## Supplementary Materials

The following supporting information can be downloaded at: https://www.mdpi.com/article/10.3390/vetsci13090927/s1, Table S1: Primer and oligonucleotide information utilized in the manuscript. Supplementary File S1: Western Blot Images.

## Abbreviations

Adenosine 5′-monophosphate (AMP)-activated protein kinase (AMPK), Duroc × Landrace × Yorkshire (DLY), fibronectin type III domain containing 5 (FNDC5), fibroblast growth factor 21 (FGF21), Fos proto-oncogene, AP-1 transcription factor subunit (FOS), glucose transporter type 4 (GLUT4), glycoprotein 130 (gp130), interleukin-6 (IL-6), interleukin-6 receptor (IL-6R), intramuscular fat (IMF), insulin-like growth factor 1 (IGF1), Janus kinase (JAK), MYC proto-oncogene, bHLH transcription factor (MYC), myocyte enhancer factor 2A (MEF2A), myocyte enhancer factor 2B (MEF2B), myocyte enhancer factor 2C (MEF2C), myostatin (MSTN), myosin heavy chain (MyHC), myosin heavy chain 4 (Myh4), myosin heavy chain 7 (Myh7), peroxisome proliferator-activated receptor gamma coactivator 1-α (PGC-1α), phospho-STAT3 (p-STAT3), satellite cells (SCs), signal transducer and activator of transcription 3 (STAT3), soluble interleukin-6 receptor (sIL-6R), suppressor of cytokine signaling 3 (SOCS3), tumor necrosis factor-α (TNF-α).
