# Supplementary material for: Myogenic IL-6 Mediates Myofiber Type Transition in Porcine Skeletal Muscle Satellite Cells via the STAT3/myh7 Pathway
Source: Vet Sci. 2026 Sep 9;13(9):927. doi: 10.3390/vetsci13090927 (PMC13612036; doi:10.3390/vetsci13090927)
Supplement: Supplementary file 1 [file vetsci-13-00927-s001.zip › Supplementary Table S1.pdf]

Table S1 Primer and oligonucleotide information utilized in the manuscript.

Name	Sequence (5'-3')	Product length (bp)	Tm (°C)	Purpose
FGF21 qF	GACTGTGGGTCCCTGTGCTG	120	60.5	For qPCR detection
FGF21 qR	CATCCGTGTAGAGGTATCGTTGG			
FNDC5 qF	TCGTGGTCCTGTTCATGTGG	125	60	For qPCR detection
FNDC5qR	CGGTGTGCTGGTTTCTGATG			
GAPDH qF	GTTCCAGTATGATTCCACCCAC	270	60	For qPCR detection
GAPDH qR	TTCACGCCCATCACAAACAT			
GLUT1 qF	CATCCTCATCGCCCAGGTATTT	168	60.5	For qPCR detection
GLUT1 qR	CGGTTCTCCTCATTGCGGTT			
HDAC5 qF	GGGCAAGGTTCTCATCGTG	119	60	For qPCR detection
HDAC5 qR	AGAAGTTCCCATTGTCATAGCGA			
IGF1 qF	CTTGGCCCTGTGCTTGCT	177	61	For qPCR detection
IGF1 qR	TCCACGATGCCCGTCTGT			

IL6 qF	CCCACCAGGAACGAAAGAGA	82	60	For qPCR detection
IL6 qR	CTGGACTGAAGGCGCTTGT			
MEF2A qF	GCCAACCGCCTACAACACC	180	60	For qPCR detection
MEF2A qR	AAATTCGAACCCTGAGATAGCTG			
MEF2B qF	CACCGACATCCTCGAGACAC	196	60	For qPCR detection
MEF2B qR	GGCCCCATATACCATGTCCG			
MEF2C qF	CAGACATCGTGGAGACGTTGA	160	60	For qPCR detection
MEF2C qR	GCACACAATCTTTGCCTGCT			
MSTN qF	CTGAATGAGAACAGCGAGCAA	140	59.5	For qPCR detection
MSTN qR	GCTGTTTCCAGGCGAAGTTTAC			
STAT3 qF	AGGACATCAGCGGTAAGACC	228	60	For qPCR detection
STAT3 qR	CACTACCTGGGTCAGCTTCG			
TNFA qF	CCACCACGCTCTTCTGCCTA	164	60	For qPCR detection
TNFA qR	CTCGGCTTTGACATTGGCTAC			

Myh4 qF	TGGTACAAAGAAAGGTGGC	150	60	For qPCR detection
Myh4 qR	CATTTAGAACCTTGTATCTCTGTT			
Myh7 qF	AAGGGCTTGAACGAGGAGTAG	110	60	For qPCR detection
Myh7 qR	TATTCTGCTTCCTCCAAAGGGG			
ChIP-Myh7-1F	CTCCCAAGCATCCTTTGTGC	147	60	For ChIP-PCR detection
ChIP-Myh7-1R	GAGACCTCAGCAACTGGCAA			
ChIP-Myh7-2F	AGGAGTGCGGCATCGG	184	60	For ChIP-PCR detection
ChIP-Myh7-2R	CTCTTGTATTCTGTGGGGTGGT			
ChIP-Myh7-3F	GCAGCTGTATGATCTGTGGC	103	60	For ChIP-PCR detection
ChIP-Myh7-3R	CCGGCCTCCGCAAAAGTA			
Myh7 Pro F	CGAGCTCTTACGCGTGCTAGCGACTCTGTGGCTGCCC	1236	60	For getting promoter region
Myh7 Pro R	CCAACAGTACCGGAATGCCATATTTTGGAGCCCTTTGCCTGT			
Myh7 Mut1 F	TTTAGATCCAAGGGGCTGGAGAGGGTGGATGAAAA	/	65	For mutating the STAT3 binding motif within

Myh7 Mut1 R	AGCCCCTTGGATCTAAAGCCACTGCCCGTCCC			Myh7 promoter
Myh7 Mut2 F	GGCTCATGAAAGGACAAGAGCTCTTTCCTGTC CCTCT	/	65	For mutating the STAT3 binding motif within Myh7 promoter
Myh7 Mut2 R	TTGTCCTTTCATGAGCCTCAGGGGCAGCCACA			
Myh7 Mut3 F	GCAACATCGAAGGGACAGGCAAAGGGCTCCA AA	/	60	For mutating the STAT3 binding motif within Myh7 promoter
Myh7 Mut3 R	TGTCCCTTCGATGTTGCCCACAGGTAGTAAATA CCCTA			
STAT3-CDS F	CCCAAGCTTATGGCCCAATGGAATCAGCTACA	2313	68	For getting CDS region
STAT3-CDS R	CCGCTCGAGTCACATGGGGGAGGTAGCG			
Si-NC-S	UUCUCCGAACGUGUCACGUTT	/	/	For knockdown control
Si-NC-AS	ACGUGACACGUUCGGAGAATT			
Si-STAT3-S	GCUAUAAGAUCAUGGAUGCUADTDT	/	/	For knockdown
Si- STAT3-AS	UAGCAUCCAUGAUCUUAUAGCDTDT			
